# Optimized polarization-independent Chand-Bali nano-antenna for thermal IR energy harvesting

**DOI:** 10.1038/s41598-023-43709-3

**Published:** 2023-10-16

**Authors:** Ahmed Y. Elsharabasy, Mohamed H. Bakr, M. Jamal Deen

**Affiliations:** 1 Engineering Mathematics and Physics Department, Faculty of Engineering, Cairo University, Giza, 12613 Egypt; 2 Electrical and Computer Engineering Department, McMaster University, Hamilton, ON L8S 4K1 Canada

**Keywords:** Devices for energy harvesting, Nanophotonics and plasmonics, Metamaterials

## Abstract

A novel, polarization-independent, wide-angle reception Chand-Bali nano-antenna is proposed. An adjoint-based optimization algorithm is used to create the same resonance at both linear polarizations of the incident radiation. The nano-antenna optimal parameters reveal that two hot spots with a strong field enhancement are created. These hot-spots could be integrated with metal–insulator–metal (MIM) diodes to form a rectenna for infrared (IR) energy harvesting. The metallic resonators allow for selecting several materials to facilitate the fabrication of the nano-antenna and the MIM diode. The Chand-Bali-based IR rectennas are investigated and simulations demonstrate an improvement of more than one order of magnitude in efficiency compared to ones using traditional nano-antennas.

## Introduction

Recent energy-scavenging technologies are attempting to mitigate the effects of decades of use of fossil fuel on our planet for future generations. These effects have stimulated the exploration of new sustainable and clean energy resources. The increasingly rapid-pace advances in internet-of-things (IoT)^1,2^ and the ubiquitous use of smart sensors and devices^3,4^ require new techniques to power them. Solar energy is considered one of the abundant and clean resources on earth. The current Si-based photovoltaics absorb the energy of photons in the visible range and convert it into DC voltage^5,6^. Several innovative attempts using different semiconductor compounds have been explored to improve the conversion efficiency of solar cells^7–12^. However, almost half of the solar spectrum, which lies in the infrared (IR) region, is not yet fully exploited^13^. Due to Planck’s theory of a black body radiation, any object above absolute zero temperature will emit IR radiation at certain wavelength corresponding to its temperature^14^. Therefore, thermal heat radiation can be considered as an unlimited energy source spreading over the IR wavelengths range from 1.0 to 10 μm. The longer wavelength of 10 μm, which is equivalent to 30 THz frequency, represents the IR radiation from objects at room temperature.

Many studies have investigated the possibility of harvesting energy at around this 10 μm wavelength^15–22^. In 1972, a smart device called rectenna (rectifying antenna) was proposed to harvest the solar energy and convert it into DC current^23^. This rectenna (antenna plus rectifier) can be described as an antenna that receive the incident electromagnetic radiation. The antenna is then connected to a rectifier which converts the captured AC current into a DC one. Recent research on rectenna prototypes have achieved quite high efficiencies > 80% in the microwave range^24–27^. However, the equivalent rectennas at IR frequencies still suffer from inadequate rectification performance^17,22^. The rectenna performance is essentially measured through the performance of each single element in the rectenna: the antenna and the diode^28^. In addition, the coupling between the two elements is considered a critical parameter in determining the total rectenna efficiency^29^. The ultra-high frequencies of the IR radiation restrict the type of diode that can be used^30^. The switching speed of the diode depends on its corresponding conduction mechanism. Owing to tunneling^27^ being the dominant conduction mechanism in metal–insulator–metal (MIM) structures, MIM diodes are considered the best candidate to operate at these ultra-high frequencies^30–32^. MIM diodes consist of two metallic layers sandwiching an insulator layer. This insulating layer must be ultra-thin, in the range of a few nanometers to maintain the fast-switching performance. Additionally, other figures-of-merit of the diode are determined from its current–voltage characteristics^33^. The most important performance measures are the diode’s resistance and responsivity^29^. The MIM diode’s resistance can vary within the range of several hundreds to Mega Ohms^34^. This resistance has to be match that of the antenna to allow for maximum power transfer. The diode’s responsivity, which is a measure of the diode’s nonlinearity, determines the rectification capability of the MIM diode^34^. Several studies and experiments were carried out to improve the diode’s performance^29^. These attempts^35–42^ were either through selecting different materials, i.e. metals and insulators, with different thicknesses, or by investigating stacks of multiple insulator layers. The main objective is still to tailor the energy band diagram of the diode to control its I–V characteristics and accordingly the diode’s resistance and responsivity. Nevertheless, the fabrication of a few-nm insulator layer(s), which is uniform and reproducible, is a crucial element for the performance of MIM diodes^16,17^. Geometric diodes based on ballistic transport theory such as graphene diodes^43,44^ were reported to achieve lower capacitance and higher rectification efficiency. Fabrication and temperature sensitive operation are among challenges that encounter this promising technology.

For a specific MIM diode, the diode’s resistance forms a constraint in selecting the antenna. In order to achieve good matching between the diode and the antenna, both resistances should be equal^29^. The dimensions of the resonant antennas are proportional to the operating wavelength in the microwave, IR, or optical regimes^45,46^. Many nano-antennas structures were proposed in literature to operate around 10 μm including nano-dipoles^47^, bowties^16,17^, spiral^48^, nano-crescent^49^, tapered dipole^50^, and log-periodic^51^. These designs resulted in an antenna resistance in the range of a few tens of Ohms^51^. This mismatch with the MIM diode’s resistance reduces the coupling efficiency. Also, the unpolarized nature of the IR radiation favors dual-polarized antenna structures. Previous work suggested the use of two linearly polarized nano-antennas such as crossed diploes^52^ or crossed bowties^53^ to overcome that requirement. Although these designs are theoretically viable, their fabrication is complex. Their coupling to diodes is also difficult. In addition, the need for a wide-angle reception is important for the nano-antenna to receive the diffusive IR radiation.

In this study, we propose a novel Chand-Bali nano-antenna design that operates around 10 μm. The proposed nano-antenna is designed using two gold metallic patches placed over a TiO_2_ substrate. A ground metallic plane was added to block transmission through the structure. A metal–insulator–metal structure is thus formed which supports magnetic resonance. This magnetic resonance allows for a wide incident-angle reception^54^. The proposed Chand-Bali nano-antenna can efficiently receive the dual-polarizations IR radiation. To improve the reception capabilities, an adjoint-based optimization algorithm was used^55^. After a few optimization iterations, we achieved a nano-antenna design that is capable of receiving the incident IR radiation at either polarization with almost no reflected power at resonance. This nano-antenna offers the opportunity to double the efficiency of IR rectennas using the traditional nano-antennas with single-polarization operation. Moreover, the calculated antenna resistance is more than twice the one in literature which provides further improvement of the coupling between the nano-antenna’s and the MIM diode. Also, the achieved two symmetric hot spots exhibit a very strong electric field enhancement. This intensive field confinement assists the tunneling mechanism through the MIM diode, which in turn boosts the rectification performance.

### The proposed Chand-Bali nano-antenna

**Figure 1:**
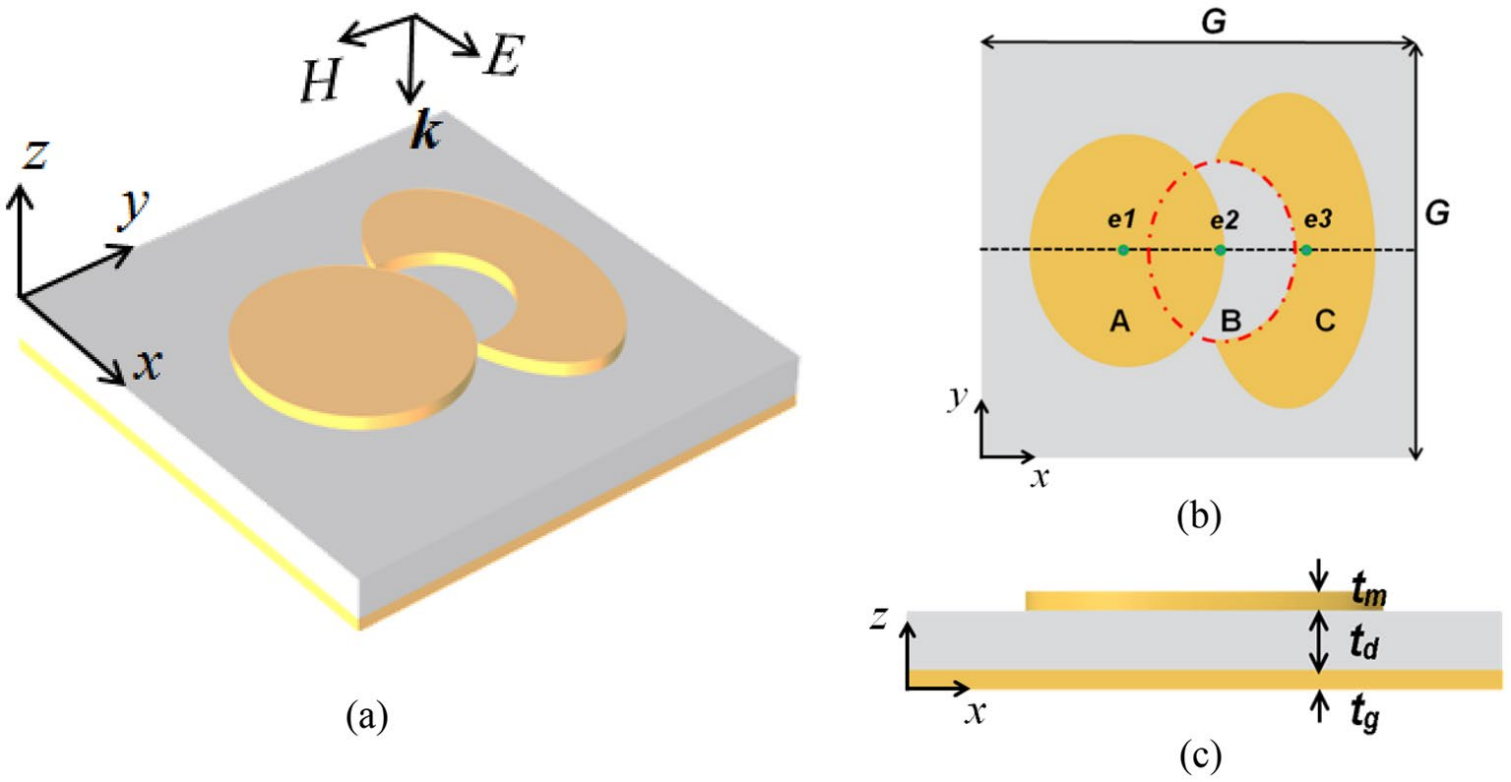
The proposed Chand-Bali nano-antenna structure: (**a**) 3D isometric view; (**b**) top view showing the design parameters of the three ellipses A, B, and C; and (**c**) cross-sectional view of the Chand-Bali nano-antenna showing the metal–insulator–metal (MIM) 3-layers whose thicknesses are design parameters.

Our design is composed of two elliptically shaped metallic patches. The first elliptic patch is designed to have its major radius along a certain direction. The second elliptic patch is cut by a smaller elliptic shape and this cut-ellipse has its major axis aligned perpendicular to the direction of the major axis of the first ellipse. From this preliminary configuration, it is possible for the nano-antenna to couple to the incident radiation with different polarizations. Figure 1 shows the structure of the proposed Chand-Bali nano-antenna. The nano-antenna is built with gold elliptic patches on top of a gold ground plane to prevent further transmission of the incident electromagnetic radiation. A thin TiO_2_ insulator layer is sandwiched between the two metals. This design, as shown in Fig. 1a, forms a metal–insulator–metal (MIM) structure. Figure 1b shows the design parameters of the proposed Chand-Bali nano-antenna. As shown in the top view, 3 different ellipses—A, B, and C are characterized by the locations of their centers and their minor and major radii. The centers *e*_1_, *e*_2_, and *e*_3_ are placed on the same axis. The developed Chand-Bali nano-antenna was assumed to lie in a periodic structure in the *x–y* plane with symmetric periodicity *G* as shown in Fig. 1b. The thicknesses of the layers (*t*_*m*_, *t*_*d*_ and *t*_*g*_) of the MIM structure are considered additional design parameters (see Fig. 1c). The thickness of the ground plane *t*_*g*_ is kept fixed at 200 nm which is several times the skin depth at the suggested operating frequency of 30 THz. The expected magnetic resonances are due to the orientation of each elliptic patch as well as their major and minor radii.

The proposed Chand-Bali nanoantenna exhibits the distinct advantage of dual-polarization operation with two open terminals. This unique combination of characteristics provides an excellent opportunity for seamless integration into both parallel and series networks, consequently enhancing the overall harvesting performance. Table 1 presents a comparative analysis of various nano-antennas reported in the literature, considering their dual polarization capabilities, number of antenna terminals, and the materials utilized in their respective designs.

**Table 1:** A comparison between different nano-antennas presented in the literature and the Chand Bali nano-antenna.

Shape	Material	Linear polarization	Terminals #	# Frequency bands	Antenna layers
Rhombic dipole^56^	Gold	Dual	4	1	1
Modified crossed dipole^57^	Gold + ITO	Dual	4	1	1
Slot dipole^58^	Gold	Single/dual	2	1	1
H-shape^59^	Silver	Dual	4	3	2
Dipole^60^	Gold	Single	2	1	1
Bowtie^17^	Gold + titanium	Single	2	1	1
Log-spiral^61^	Gold + silver	Single	2	1	1
Arrow Bowtie^62^	Graphene	Single	2	1	1
Chand Bali (this work)	Gold	Dual	2	1	1

All nano-antennas work around ~ 30 THz (~ 10 µm) for energy harvesting applications.

### The adjoint-based optimization

As described in the simulation steps, two different simulations were performed to determine the reflectance at each polarization. However, calculating the electric field enhancement at the gap is critical. ANSYS HFSS can be used to calculate the scattering (*S*-) parameters in addition to their derivatives with respect to the geometry and material parameters. These derivatives are estimated using a self-adjoint method with no additional simulations^55^. Therefore, seeking a strong electric field confinement in both polarizations simultaneously can be defined as an optimization problem. Gradient-based optimization algorithms generally require fewer iterations and hence simulations, as compared to global optimization methods. The gradient of the electric field is not possible through the self-adjoint method available in ANSYS HFSS. This implies that a huge number of simulations are required to approximate the gradient using finite difference methods, for example, especially in the case of many design parameters. Therefore, the need for dealing numerically with the *S*-parameters is crucial.

The required link between *S*-parameters and the electric field enhancement can be derived through the coupled-mode theory (CMT)^63^. In CMT, the optimized field enhancement of a given nano-antenna with a specific material is directly proportional to the absorption quality factor *Q*_abs_. This optimum quality factor occurs at the reflectance valleys^64^. Thus, the field enhancement is associated with minimum reflectance wavelengths. An optimization algorithm can be used to minimize the reflectance using:

1 $$\begin{gathered} {\text{Reflectance = }}\left| {S_{11} } \right|^{2} , \hfill \\ \hfill \\ \end{gathered}$$

2 $$\, W = {1} - \left| {S_{11} } \right|^{2} \propto \, \left| {\frac{E}{{E_{0} }}} \right|^{2} ,$$

where *E*_0_ is the incident electric field and *W* is the objective function. The optimization problem can be formulated as,

3 $$\mathop {\max }\limits_{u} \, (W_{1} ({\mathbf{u}},\lambda ) + W_{2} ({\mathbf{u}},\lambda )), \, \lambda = {10}\,\upmu {\text{m, }}{\mathbf{c}}{(}{\mathbf{u}}{)} \le {0,}$$

where *W*_1_ and *W*_2_ are the reflectance calculated for an incident electromagnetic wave with electric field polarized in *x* and *y* directions, respectively, at a wavelength of 10 μm. The vector ***c*** represents the linear and nonlinear geometrical constraints to avoid non-physical structures. The design parameters ***u*** is determined from the geometries as shown in Fig. 1b,c, where,

4 $${\mathbf{u}} = [G, \, r_{Ax} , \, r_{Ay} , \, r_{Bx} , \, r_{By} , \, r_{Cx} , \, r_{Cy} , \, e_{1} , \, e_{2} , \, e_{3} , \, t_{m} , \, t_{d} ]^{T} .$$

These 12 design parameters are categorized into three classes: The unit cell periodicity (*G*), the thicknesses of the top metals and the insulator layer (*t*_*m*_, *t*_d_) respectively, and finally the major and minor radius of each ellipse (*r*_*x*_, *r*_*y*_) and their center locations *e*_*i*_.

**Figure 2:**
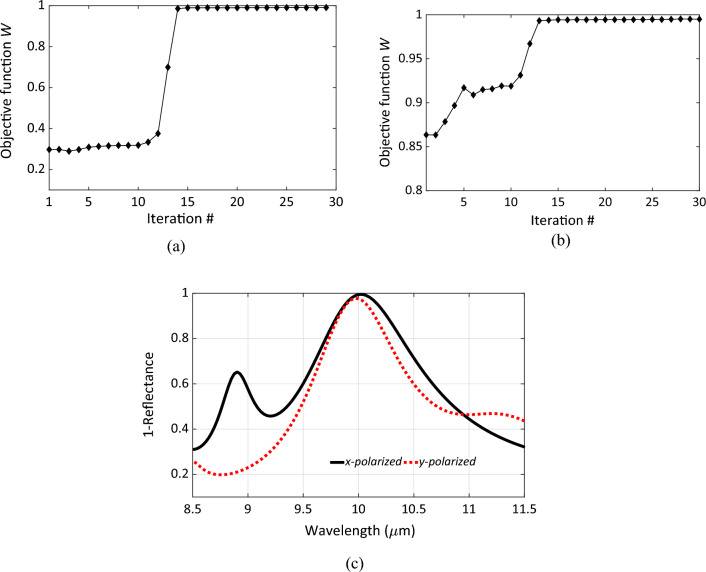
(**a**) The convergence of the optimization algorithm measured with the objective function (*W* = 1 − reflectance) versus the iteration number; the algorithm approaches the maximum absorptivity after 15 steps for the case of *x*-polarized incident E-field. (**b**) The convergence of the algorithm in the case of two parallel simulations with electric field polarizations are in *x* and *y* directions, respectively. (**c**) The absorbance (= 1 − reflectance) of the Chand-Bali nano-antenna calculated at optimal design parameters for *x*-polarized incident electric field (solid), and *y*-polarized incident electric field (dotted).

Attempting to simultaneously minimize the reflectance of both polarizations did not yield a good design. Therefore, the optimization problem is updated to obtain a starting feasible point. First, the optimization is carried out for *W*_1_ only to obtain an optimal point for the first polarization. As shown in Fig. 2a, the convergence of this optimization step is achieved after 15 iterations. These optimal design parameters are then used to perform the optimization step for both polarizations simultaneously as described in Eq. (3). This starting point, which is optimal for one specific polarization, is not optimal for the other one, as shown in Fig. 2b. The second optimization step started from an initial reflectance of (1 − 0.86) = 0.14 and achieved a reflectance of less than 0.01 after 13 iterations. The achieved design minimizes the reflectance for both polarizations as shown in Fig. 2c. Both peaks are very close to a unity value at wavelength of 10 μm. The optimization algorithm is performed in a MATLAB environment with tailored scripts to link ANSYS HFSS and automate the process.

## Results and discussions

### The field enhancement factor and field distribution

**Figure 3:**
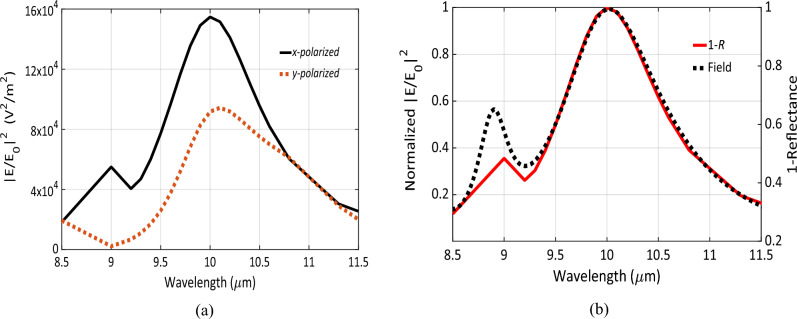
(**a**) The enhancement of the electric field intensity |E/E_0_|^2^ of the optimal design of the Chand-Bali nano-antenna structure vs. wavelength, the *x*-polarized is in solid while *y*-polarized is plotted as dotted lines. (**b**) The normalized enhancement factor for an *x*-polarized incident wave plotted with (1 − Refelctance) of the same *x*-polarized one showing matching response around the resonance wavelength.

Numerical simulations are carried out using the optimal set of design parameters obtained from executing the optimization algorithm. These optimal dimensions are presented in Table 2. The electric field at the center of each gap was simulated over the wavelength range from 8.5 to 11.5 μm (see Fig. 3a). A strong electric field confinement is noticed at 10 μm for both polarizations. The gap in the optimal design is 15 nm, which can be fabricated using electron beam lithography (EBL)^16,17^. The electric field enhancement factor approaches 1.5 × 10^5^ and ~ 10^5^ for the *x* and *y* polarized incident electromagnetic waves at 10 μm, respectively. It is expected to have different enhancement factor for different polarizations as the nano-antenna is not symmetric. However, both polarizations support the antenna’s resonance at 10 μm. The small peak at shorter wavelength as shown in Fig. 3a can be attributed to surface plasmon resonance (SPP) supported by the nano-antenna array^9^. In Fig. 3b, the electric field enhancement in the case of the *x*-polarized EM wave was normalized and plotted with the corresponding reflectance from the *S*-parameters. Both curves are identical around the resonance, thereby validating the assumptions from coupled-mode theory.

**Table 2:** The optimal dimensions of the Chand Bali nano-antenna.

Parameters	*G*	*r*_*Ax*_	*r*_*Ay*_	*r*_*Cx*_	*r*_*Cy*_	*r*_*Bx*_	*r*_*By*_	*e*_*1*_	*e*_*2*_	*e*_*3*_	*t*_*d*_	*t*_*m*_
Optimal values [nm]	7040	1498	1612	1220	2188	1088	1030	843	1410	450	600	204

**Figure 4:**
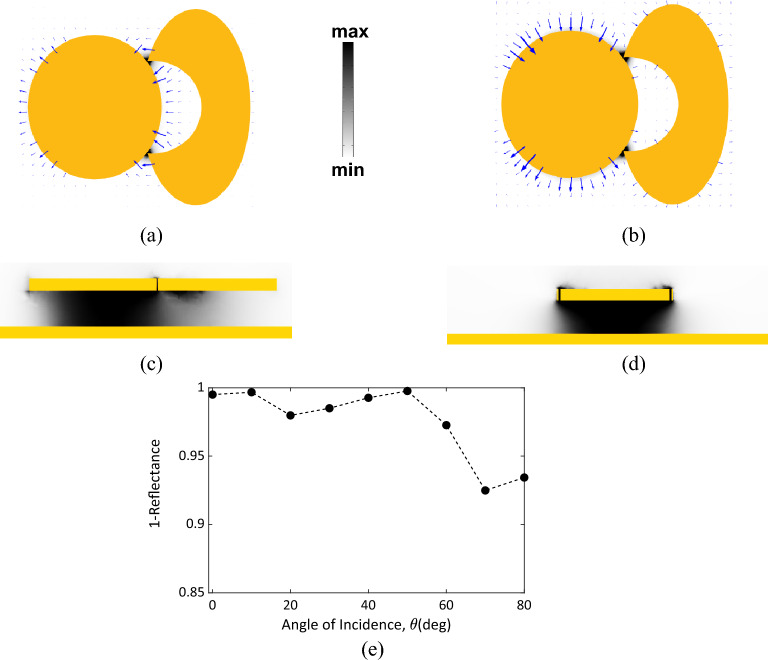
The distribution of the electric field intensity |E|^2^ of the optimal design of the Chand-Bali nano-antenna structure calculated at 10 μm. (**a,b**) At the center *xy*-plane, where the darker color represents higher electric field intensity, all plots at the same scale, and the arrows represent the electric field vector at the same resonance wavelength of 10 μm for (**a**) *x*-polarized incident electric field, and (**b**) *y*-polarized incident electric field, respectively. (**c,d**) The grey scale spectrum maps the magnetic field intensity cross-sections emphasizing the creation of magnetic resonances. The cross-section is cut parallel to *xz-*plane passes through the hot-spot gap in (**c**), while it is parallel to *yz-*plane and passes through the two hotspots in (**d**). (**e**) The reflectance at the resonance wavelength with varying the incident angle, *θ*, the absorbance is over 92% for incident angles up to 80°.

The electric field distribution over the *xy*-plane at the resonance wavelength was calculated and is plotted in Fig. 4. When a normal incident wave impinges the nano-antenna with electric field polarized along the *x*-axis at resonance, the electric field will be confined across the two gaps. These confinements form two hot spots which support the operation of the MIM diode to rectify the harvested fields. The electric field vectors shown in Fig. 4a reveal that the charges on the elliptic patch on the right is divided into two longitudinally opposite polarities to support this resonance mode. When the incident electric field is vertically polarized along the *y*-axis, the charges over the elliptic patch are split between the upper and lower halves with opposite polarity to allow for the corresponding resonance as shown in Fig. 4b.

The magnetic field distribution of the *x*-polarized incident wave is plotted in cross section passing through the hot-spot and parallel to the *xz*-plane as shown in Fig. 4c. The magnetic field distribution across the *yz*-plane for the *y*-polarized incident wave is presented in Fig. 4d. Both magnetic field distributions exhibit magnetic resonance at 10 μm^54^, which in turn allow for a wide-angle performance for oblique incidence. The reflectance is calculated with variable incident angle *θ*, at the resonance wavelength of 10μm and presented in Fig. 4e. The absorbance is over 92% for incident angles up to 80°. This important feature proves that the proposed Chand-Bali nano-antenna is one of the most competitive energy harvesters for the diffusive IR radiation.

### MIM diodes and rectenna efficiency

The rectenna in the IR region consists of a nano-antenna connected to a diode. The nano-antenna receives the IR radiation with wavelengths matched to the resonance wavelength of the nano-antenna. This collected ultra-high frequency AC signal is then passed through the diode to be rectified and produces a useful DC current. The proposed optimal Chand-Bali nano-antenna possesses a high electric-field enhancement at the designated gaps to assist and improve the diode’s performance. Absorbing IR radiation with a very wide range of incident angles further boosts the rectenna performance. In spite of these merits, the impedance matching with the diode can be a challenge to the performance of the rectenna^29^. MIM diodes can theoretically operate up to visible frequencies^22^. However, one crucial concern is that the diode’s high nonlinearity is generally associated with a large resistance^34^. This resistance varies from hundreds to Mega Ohms^34^. This huge difference with the resistance of the nano-antenna can prevent the highly efficient nano-antennas from delivering the collected power to the diode, which in turn would make the rectenna inefficient^17^. One of the solutions to resolve this conflict is to build nano-antennas with high impedance in order to mitigate the mismatching effects.

**Figure 5:**
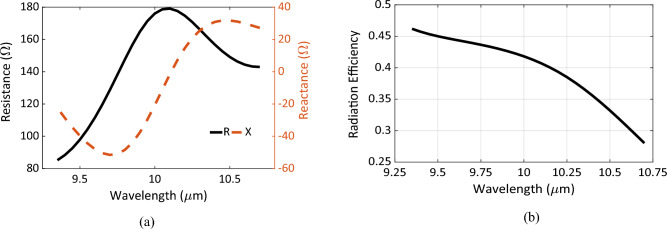
(**a**) The impedance of the Chand-Bali nano-antenna structure calculated around 10 μm, the resistance *R* in solid, and the reactance *X* in dashes, (**b**) the radiation efficiency of the optimal Chand-Bali nano-antenna calculated around the FWHM around 10 μm.

The optimal Chand-Bali antenna is then numerically simulated in the transmission mode by defining a lumped port in one of the gaps with a matched lumped load at the other gap. The antenna’s far-field analysis is carried out to estimate the far-field patterns and antenna parameters. The full-width-half-maximum (FWHM) of the proposed nano-antenna can be derived from Fig. 3b, and it is calculated to be from 9.3 to 10.7 μm. The simulations were carried out at the wavelength range of the FWHM. The performance of the nano-antenna outside this range is significantly attenuated due to poor coupling with the diode. Figure 5a represents the nano-antenna impedance calculated at the FWHM range.

The impedance matching efficiency *η*_*m*_ for an MIM diode with resistance *R*_*d*_, and a nano-antenna resistance *R*_*a*_ can be formulated as^25^:

5 $$\eta_{m} = \frac{{4R_{a} R_{d} }}{{(R_{a} + R_{d} )^{2} }}.$$

The resistance at the resonance wavelength of 10 μm is ~ 180 Ω, which is more than 3 times that of the fabricated bow-tie nano-antenna at this wavelength^17^. This is reflected on the matching efficiency improvement by almost the same factor. Also, it is noticed that the reactance part of the nano-antenna impedance should be taken into consideration in computing the matching or coupling efficiency. The integration should include both parts in the calculations in order to avoid inaccurate efficiencies.

The nano-antenna radiation efficiency was computed and is presented in Fig. 5b. The radiation efficiency is almost 43% at resonance, which is ~ 4 times that of the bow-tie nano-antenna described in Ref.^13^. The whole efficiency is double this value as the design can receive both polarizations simultaneously. The rectenna efficiency *η*_Rec_ is approximated using the following formula:

6 $$\eta_{{{\text{Rec}}}} = \eta_{a} \times \eta_{s} \times \eta_{c} \times \eta_{j} ,$$

where *η*_*a*_ is the nano-antenna efficiency related to the ability of the nano-antenna to collect the incident electromagnetic radiation, *η*_*s*_ is the efficiency of transferring the collected energy by the antenna to the diode terminals, *η*_*c*_ is the coupling efficiency between the antenna and the diode and *η*_*j*_ is the efficiency of rectifying the AC power through the diode. The last term (*η*_*j*_) can be determined by measuring the diode’s responsivity. The coupling efficiency is proportional to the matching efficiency^29^. Therefore, the overall efficiency is likely to be boosted by three main factors. There is a dual-polarization operation which is reflected as approximately two times increase. Also, a larger nano-antenna resistance leads to the second increase with almost the same factor due the rise in the matching efficiency. Therefore, a rise of more than three times in the coupling efficiency compared to the bow-tie nano-antenna is achieved. Finally, the proposed nano-antenna has a radiation efficiency close to 4 times higher than results reported from bow-tie-based IR rectennas. These three factors successfully achieve more than one order of magnitude improvement in the rectenna’s overall efficiency.

### Fabrication considerations

The proposed Chand-Bali nano-antenna offers two space gaps which facilitate the fabrication of the diode by considering that the antenna’s metallic patches also function as the two metallic sides of the MIM diode. However, from the diode characteristics and figures-of-merit, it is preferable to build the MIM diode with different metal electrodes rather than use the antenna’s metal layers^33,34,65^. The difference in the work function between the two metals offers an opportunity to improve the diode’s responsivity^29^. Therefore, the metallic cut-elliptic patch, which was initially designed of gold, was replaced by a titanium one. The work functions for gold and titanium are 5.1 eV and 4.33 eV, respectively, which is expected to increase the diode’s rectification capability. Also, Ti is well known in forming a thin oxide layer when exposed to air, which in turn simplify the fabrication of the insulator layer to form the diode. However, one drawback is that the oxide layer grows in all possible directions, and as a result, the nano-antenna top layer may also have a TiO_2_ layer on top. The final design would take the shape of a large array with parallel and series connections of each antenna, where each antenna cell has a periodicity of 6.5 µm in both directions.

**Figure 6:**
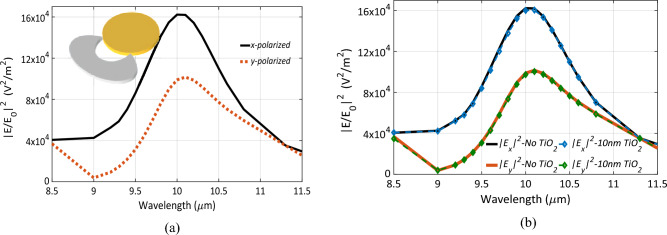
(**a**) The enhancement of the electric field intensity |E/E_0_|^2^ of the optimal design of the Chand-Bali nano-antenna structure vs. wavelength, with the cut-ellipse is designed from Ti instead of gold, while the other elliptic patch is still in gold the *x*-polarized is in solid, the inset shows the new nano antenna structure after changing the materials. (**b**) The field enhancement with the Ti–Au patches as in (**a**) with the difference of adding a thin layer of TiO_2_ over the Ti patch, the solid lines represent the case of no oxide layers, and the dashed lines with diamond symbols attributed the response after adding the 10 nm oxide layer, both cases show perfect fit.

Figure 6a shows the electric field enhancement calculated to study the case of changing the material of the cut-elliptic patch from gold to titanium. The enhancement factor improved slightly by comparing peaks in Figs. 3a and 6a. However, a slight red-shift occurred for both polarizations which is attributed to the different complex permittivities of gold and titanium at this wavelength range. The effect of adding a 10 nm-thick layer of TiO_2_ on the top of the Ti patch was investigated. The simulations showed almost no change in the performance of the nano-antenna in both cases as presented in Fig. 6b. This insensitive performance reveals the feasibility of the proposed Chand-Bali nano-antenna in IR rectenna design under normal practical conditions.

The ground plane can be fabricated from Ti instead of Au as this allows for easy growth of the TiO_2_ oxide layer of the substrate. There is also a possibility to form a single-insulator layer MIM diode or multiple-insulator layers such as MIIM diode to improve the overall performance by considering the gap separations between both ellipses in the range of few nanometers^64^. The proposed nano-antenna design combined with the optimization algorithm offer a flexible and scalable way to build energy harvesters operating at a specific wavelength or a narrow wavelength range. The sharp tips of the proposed nano-antenna were rounded in the simulations and showed insensitive response for the absorbance capabilities at the resonance wavelength. Also, the radii of ellipses were varied to simulate the fabrication tolerances at this nanometer scale and resulted in insignificant shift in the resonance wavelength.

### MIM diode analysis

**Figure 7:**
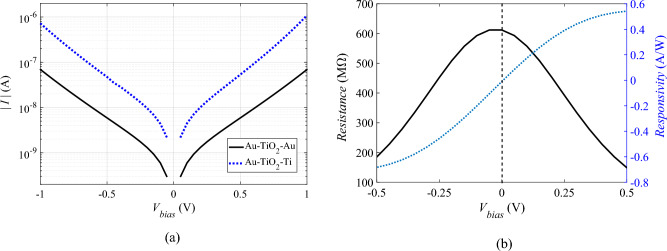
(**a**) The current–voltage characteristics of the MIM diode, the Au–TiO_2_–Au based in solid lines while the Au–TiO_2_–Ti based in dotted. (**b**) The resistance and responsivity of the Au–TiO_2_–Au based MIM diode.

The metal–insulator–metal (MIM) diodes are suitable candidates that can work with the proposed nanoantenna in the IR region. The dominant tunneling current through thin oxide layers with a few nanometers thickness enables the MIM diodes to rectify the ultra-high frequency AC signal received from the nanoantenna. Furthermore, the fabrication of the nanoantenna integrated with an MIM diode would reduce the complexity by designing the metallic ground plane made of titanium instead of gold. Also, one arm of the nanoantenna is designed to be made from titanium instead to improve the MIM diode asymmetry as shown in Fig. 6a. The estimated current^34,64^ from MIM diode is plotted in Fig. 7a. The different metallic electrodes show more asymmetric behavior as expected and illustrated in Fig. 7a. The resistance and responsivity of the Au-TiO_2_-Au based MIM diode are calculated from the estimated I–V characteristics and presented in Fig. 7b. An improved responsivity is expected from using multiple insulator layers in building the MIM diode.

## Conclusions

A novel nano-antenna design was investigated for use in rectennas for infrared (IR) energy harvesting. The proposed Chand-Bali nano-antenna is an excellent candidate to receive randomly polarized IR radiation around 10 μm. An adjoint-based optimization algorithm was exploited to achieve maximum field enhancement at the nano-antenna gaps for dual polarizations simultaneously at the same operating wavelength. The algorithm succeeded in producing parameters for an optimal design that allow for near unity absorbance at 10 μm. The optimal Chand-Bali design possesses a strong electric field enhancement factor of more than 10^5^ at the center of gaps whose width is 15 nm. Also, the nano-antenna was developed as a metal–insulator–metal (MIM) structure. This MIM structure exhibited a magnetic resonance and as a result extended the reception capabilities efficiently for angle of incidences up to 80°. The antenna resistance was 180 Ω which improved the matching with the diode. The radiation efficiency was also computed as 43% with a maximum detectivity of 5.5. The numerical simulations for different materials were carried out with insignificant impact on the nano-antenna’s performance. The selection of metals and insulators supported connecting with several MIM diodes to improve the overall rectenna’s performance. Finally, this optimized Chand-Bali nano-antenna achieved more than one order of magnitude improvement compared with the fabricated bow-tie nano-antennas operating at the same wavelength range.

## Methods

### Numerical simulations

To quantify the performance of the proposed Chand-Bali nano-antenna, the electric and magnetic fields should be calculated under the operation conditions. Therefore, the nano-antenna was analyzed using the finite element method (FEM) solver ANSYS HFSS. COMSOL Multiphysics was used to validate the results from ANSYS HFSS. The nano-antenna was built in an air box with periodic conditions on the sides to mimic the effect of an infinite array structure. A port is set on the top of the air box to excite the nano-antenna around 30 THz with a normal incident wave. A perfectly matched layer (PML) is designed on the top of the structure as an absorbing boundary condition. Gold and titanium dioxide are modeled using their complex permittivities within the considered frequency range^66,67^. Consistent mesh parameters are selected to ensure convergence of the calculations. From the defined port, *S*-parameters are computed, and reflectance and absorbance are then determined. The simulations were repeated twice under different electric field polarizations in order to determine the corresponding performance.
